# Associations between Physical Activity, Sunshine Duration and Osteoporosis According to Obesity and Other Lifestyle Factors: A Nested Case–Control Study

**DOI:** 10.3390/ijerph18094437

**Published:** 2021-04-22

**Authors:** Chan-Yang Min, Dae-Myoung Yoo, Hyo-Geun Choi

**Affiliations:** 1 Hallym Data Science Laboratory, Hallym University College of Medicine, Anyang 14068 Korea; 42479@hallym.ac.kr (C.-Y.M.); ydm1285@hallym.ac.kr (D.-M.Y.); 2 Department of Otorhinolaryngology-Head & Neck Surgery, Hallym University College of Medicine, Anyang 14608, Korea

**Keywords:** osteoporosis, bone mineral density, physical activity, sunshine duration, vitamin D, lifestyle, obesity, smoking, alcohol consumption

## Abstract

(1) Background: The purpose of the study was to evaluate the associations between physical activity (PA), sunshine duration (SD) and the occurrence of osteoporosis according to lifestyle status. (2) Methods: Data from the Korean National Health Insurance Service–National Sample Cohort (NHIS-NSC) collected from 2009 to 2015 were used. Osteoporosis (*n* = 19,351) and control (*n* = 38,702) participants were matched in a 1:2 ratio according to age, sex, income, and region of residence. PA was classified as moderate- to high-intensity PA (MHPA) or low-intensity PA (LPA) based on the International Physical Activity Questionnaire (IPAQ). SD was classified as short (≤6 h) or long (>6 h). Conditional logistic regression was used to calculate the odds ratios (ORs) with 95% confidence intervals (CIs) of MHPA and long SD for the occurrence of osteoporosis. Subgroup analyses were performed according to SD (or PA), obesity, smoking, and alcohol consumption. (3) The adjusted OR of MHPA for osteoporosis was 0.90 (95% CI = 0.87–0.94). The results were consistent in the age/sex, SD, obesity, smoking, and alcohol consumption subgroups, but not the <60-year-old male and underweight subgroups. The adjusted OR of long SD for osteoporosis was 0.96 (95% CI = 0.93–1.00). The findings were consistent in the <60-year-old female, obese, nonsmoker, and <1 time a week alcohol consumption subgroups. (4) Conclusions: We suggest that both higher intensity of PA and long SD could decrease the risk of osteoporosis. Specifically, PA could decrease the risk of osteoporosis in individuals with most characteristics except male sex or underweight. Long SD could decrease the risk of osteoporosis in young females, obese individuals, nonsmokers, and individuals with lower alcohol consumption.

## 1. Introduction

In 2020, the novel coronavirus disease (COVID-19) outbreak occurred worldwide. This serious pandemic not only affected the burden of coronavirus disease but also impacted psychological health, reduced physical activity (PA), and caused weight gain because of the COVID-19 lockdown [1]. During the pandemic, PA has been important for enhancing the immune system and maintaining physical and mental health [2]. However, group PA in confined spaces should be restricted due to the possibility of increasing the infection rate [3,4]. Instead of indoor group PA, PA at home or outdoor PA is recommended [2]. In fact, according to up-to-date studies on COVID-19, outdoor PA, such as bicycling and walking, is increasing and is expected to be an alternative to public transportation that allows for social distancing [5,6].

The benefits of outdoor PA are not only PA itself but also increased sun exposure, which improves vitamin D status. Both benefits are effective in improving musculoskeletal conditions, especially bone mineral density (BMD) [7]. Osteoporosis is characterized by decreased BMD [8]. Preventing osteoporosis by increasing BMD is important because osteoporosis can cause fractures that lead to death in elderly individuals [9,10].

Osteoporosis is closely related to lifestyle factors such as PA, dietary intake, vitamin D status, obesity, smoking, and alcohol consumption [7]. Increasing evidence has shown that PA could improve BMD, thereby preventing osteoporosis and fractures [11,12,13]. Furthermore, previous studies, including our previous study, have investigated the combined effect of PA and other lifestyle factors, such as body mass index (BMI, kg/m^2^) or serum vitamin D, on BMD [14,15]. However, studies on the combined association of PA and other lifestyle factors, such as smoking and alcohol consumption, with bone loss are rare.

Sun exposure supplies vitamin D, which enhances bone health. Other sources of vitamin D are source foods and vitamin D supplements. Elevated serum 25-hydroxyvitamin D (25[OH]D), the form of vitamin D in the body, could increase the absorption of calcium and phosphorus from the intestine by reducing parathyroid hormone (PTH) secretion to increase BMD [16,17]. An increasing number of studies are being conducted on the association between vitamin D and BMD. However, the intake of vitamin D alone is not evident to prevent fractures [18,19]; instead, review studies have suggested that combining vitamin D with calcium intake is associated with a lower risk of osteoporosis and fractures [20,21].

On the other hand, an experimental study demonstrated that sunlight exposure is more effective for improving bone structure than vitamin D supplementation in rats [22]. One study confirmed that fracture prevalence was inversely associated with lifetime ultraviolet (UV) radiation exposure according to the Beagley–Gibson (BG) grade in women [23]. Another study from South Korea reported that the prevalence of vitamin D deficiency was higher in northern cities, which are relatively far from the equator, than in southern cities [24]. Accordingly, daily sunshine duration (SD) could increase vitamin D levels to improve bone health. However, the association between daily SD and osteoporosis in humans is still questionable.

One of our previous studies investigated whether the combination of PA and serum 25(OH)D is inversely associated with osteopenia or osteoporosis using national population survey data with a cross-sectional study design [15]. Our study also examined the association between the intensity of PA and various causes of death using longitudinal national cohort data [25]. Furthermore, the purpose of our current study was to identify the association between PA and osteoporosis and between SD and osteoporosis using the same national cohort data that were used in our previous study [25]. In addition, we performed various subgroup analyses in our study, including age/sex, SD (or intensity of PA), obesity, smoking, and alcohol consumption status.

## 2. Materials and Methods

### 2.1. Study Population and Participant Selection

This study was approved by the ethics committee of Hallym University (HALLYM 2019-08-029). The requirement for written consent was waived by the Institutional Review Board. All analyses followed the guidelines and regulations of the ethics committee of Hallym University.

Health screening cohort data from the Korean National Health Insurance Service–National Sample Cohort (NHIS-NSC) were used in this study. The details of the Korean NHIS-NSC have been described elsewhere [26]. A total of 514,866 participants who were randomly selected from 5,150,000 health insurance holders and who had undergone a health screening by NHIS from 2002 to 2003 in Korea were included. The follow-up period of the data was from 2002 to 2015.

**Figure 1:**
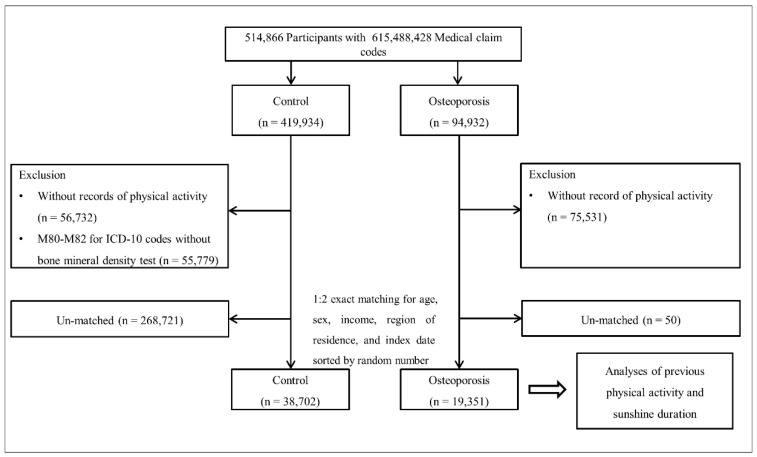
A schematic illustration of the participant selection process that was used in the present study. Of a total of 514,866 participants, 19,351 osteoporosis patients were matched with 38,702 control participants for age, sex, income, and region of residence.

Among 514,866 participants, 94,932 were included in the osteoporosis group according to the osteoporosis definition, and 419,934 were included in the control group. Participants who did not have PA records were excluded. As the PA information was different from 2002 to 2008, those participants were also excluded (*n* = 75,531 in the osteoporosis group, *n* = 56,732 in the control group). Control participants who were diagnosed with International Classification of Diseases, 10th version (ICD-10) codes M80–M82 without BMD test data were excluded (*n* = 55,779). The matching method that was previously used in our studies was conducted [27,28]. The osteoporosis and control groups were matched in a 1:2 ratio by age, sex, income, and region of residence. Control participants were sorted by random number to minimize selection bias. The index date of each osteoporosis participant was defined as the date of the onset of osteoporosis. Each control participant was assigned the same index date as each matched osteoporosis participant. During the matching process, 50 osteoporosis participants and 268,721 control participants were excluded. A total of 19,351 osteoporosis participants and 38,702 control participants were included in the final analysis (Figure 1).

### 2.2. Definition of Osteoporosis (Outcome)

Osteoporosis patients were defined as participants diagnosed with M80 (osteoporosis with pathological fracture), M81 (osteoporosis without pathological fracture), or M82 (osteoporosis in diseases classified elsewhere) using ICD-10 codes ≥ 2 times and whose BMD was tested using dual energy X-ray absorptiometry (DEXA) or computed tomography (CT) scans (Claim codes: E7001-E7004, HC341-HC345).

### 2.3. Determination of Moderate- to High-Intensity Physical Activity and Low-Intensity Physical Activity (Exposure)

PA information was collected using a modified International Physical Activity Questionnaire (IPAQ) [29]. The questionnaire asked the number of days the participants walked ≥30 min, performed moderate-intensity activity for ≥30 min, or performed vigorous-intensity activity for ≥20 min in a week. We used PA information from the first record of health screening. ‘Moderate- to high-intensity PA (MHPA)’ was defined as walking ≥5 days, performing a moderate-intensity activity ≥ 5 days, performing a vigorous-intensity activity ≥3 days, or any combination of walking, moderate-intensity activity, or vigorous-intensity activity ≥5 days with ≥600 metabolic equivalent (MET)-min/week based on the IPAQ. Other participants were classified in the ‘low-intensity PA (LPA)’ group.

### 2.4. Classification of Sunshine Duration (Exposure)

SD was defined as a measure of the hours of sunshine hours per day at each given location, excluding the duration of time it was cloudy or foggy. SD data were collected by the Korea Meteorological Administration (KMA) measured hourly by an automated synoptic observing system (ASOS) and manual measurement at 94 locations [30]. We merged SD data and NHIS-NSC data by residential area and index year. The residential areas were classified as Seoul, Busan, Daegu, Incheon, Gwangju, Daejeon, Ulsan, Gyeonggi, Gangwon, Chungcheongbuk, Chungcheongnam, Jeollabuk, Jeollanam, Gyeongsangbuk, Gyeongsangnam, and Jeju (16 areas). As NHIS-NSC data are annual data, mean values of 1 year (365 days) for SD were calculated based on the index year of participants. SD was classified into short SD (≤6 h) and long SD (>6 h) based on the median value of SD (6.0 h).

### 2.5. Covariates

The classification of age, income, and region of residence was performed as described in our previous study [25,31]. Age was collected from participants who were ≥ 40 years old in 2002 and 2003 and classified into 9 groups at 5-year intervals. The income groups were classified from 1 (low) to 5 (high) level, and region of residence was divided into urban and rural.

Tobacco smoking status (nonsmoker, past smoker, current smoker), alcohol consumption (<1 time a week, ≥1 time a week), and obesity based on BMI (<18.5 for underweight, ≥18.5 to <23 for normal weight, ≥23 to <25 for overweight, ≥25 to <30 for obese I, ≥30 for obese II) were classified in the same way as in the previous study [25,31].

To evaluate the burden of comorbidities, the Charlson Comorbidity Index (CCI) score was used. The CCI includes 17 comorbidities. Higher CCI scores indicate more severe and varied comorbidities. In our study, the CCI was scored as 0 (no comorbidities) to 15 (7 comorbidities) as a continuous variable [32].

### 2.6. Statistical Analyses

The general characteristics were compared between the osteoporosis and control groups using the chi-square test.

Conditional logistic regression was used to analyze the odds ratios (ORs) with 95% confidence intervals (CIs) for osteoporosis in the MHPA group compared to the LPA (control) group and in the long SD group compared to the short SD (control) group. In this analysis, crude and adjusted models were calculated. In the PA groups, SD, obesity, smoking status, alcohol consumption, and CCI scores were adjusted in the adjusted model. In the SD group, PA instead of SD and other covariates were adjusted in the adjusted model. The analysis was stratified by age, sex, income, and region of residence.

For the subgroup analyses, we regrouped participants by age and sex (<60 years old and ≥60 years old; males and females) and analyzed the crude and adjusted models with a conditional logistic regression model.

Additionally, subgroup analyses according to SD (short and long SD)/PA (LPA and MHPA), obesity (underweight, normal weight, overweight, obese), smoking status (nonsmoker and past/current smoker), and alcohol consumption (<1 time a week and ≥1 time a week) were performed using model 1 (adjusted for age, sex, income, and region of residence) and model 2 (adjusted for model 1 plus smoking status, alcohol consumption, CCI scores, and SD (or PA)). In these analyses, we used an unconditional logistic regression.

Two-tailed analyses were performed, and significance was indicated by a *p*-value < 0.05. We used SAS version 9.4 (SAS Institute Inc. Cary, NC, USA) for statistical analyses.

## 3. Results

The general characteristics of the participants are shown in Table 1. The prevalence of MHPA in the osteoporosis group was significantly lower than the prevalence in the control group (42.1% (*n* = 8,142/19,351) vs. 44.6% (*n* = 17,240/38,702), *p* < 0.001). The prevalence of long SD in the osteoporosis group was significantly lower than the prevalence in the control group (50.3% (9,726/19,351) vs. 51.5% (19,930/38,702), *p* = 0.005).

The adjusted OR for osteoporosis in the MHPA group was 0.90 (95% CI = 0.87–0.94, *p* < 0.001). In subgroup analyses according to age and sex, the findings were consistent with the main results in <60-year-old females and in ≥60-year-old males and females (*p* < 0.05, Table 2).

The adjusted OR for osteoporosis in the long SD group was 0.96 (95% CI = 0.93–1.00, *p* = 0.027). In subgroup analyses according to age and sex, the findings were consistent with the above finding only in < 60-year-old females (*p* < 0.05, Table 3).

**Figure 2:**
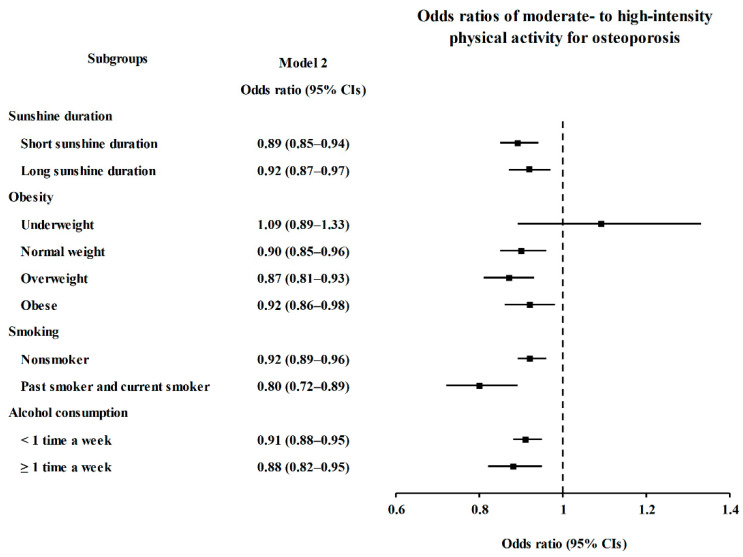
Subgroup Analyses of Moderate- to High-Intensity Physical Activity for Osteoporosis According to Sunshine Duration, Obesity, Smoking, and Alcohol Consumption Visualized by Forest Plot.

In subgroup analyses conducted in the PA groups, the ORs of MHPA for osteoporosis were significantly lower in the short and long SD, normal weight, overweight, obese, nonsmoker, past and current smoker, and <1 time a week and ≥1 time a week of alcohol consumption subgroups (*p* < 0.05, Figure 2 and Table S1).

**Figure 3:**
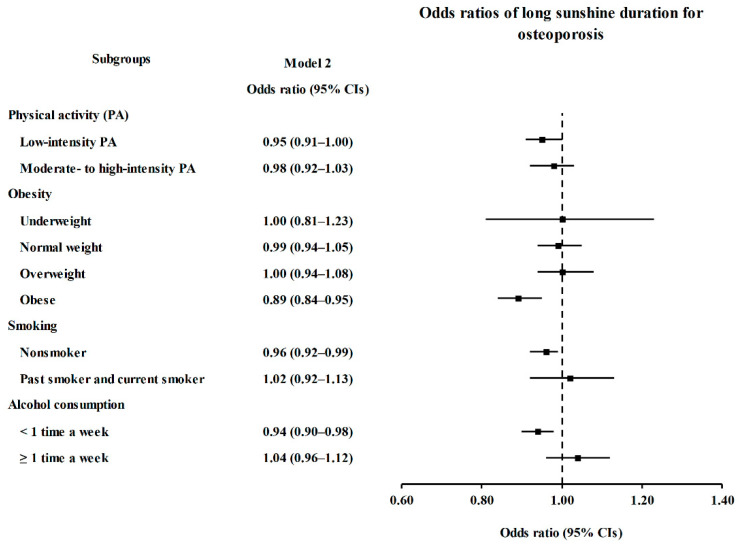
Subgroup Analyses of Long Sunshine Duration for Osteoporosis According to Physical Activity, Obesity, Smoking, and Alcohol Consumption Visualized by Forest Plot.

In subgroup analyses conducted in the SD groups, the ORs of long SD for osteoporosis were significantly lower in the LPA, obese, nonsmoker, and <1 time a week alcohol consumption subgroups (*p* < 0.05, Figure 3 and Table S2).

## 4. Discussion

We evaluated whether PA and osteoporosis, and sunshine duration and osteoporosis were associated using data from a large national cohort with 7-year follow-up periods. To the best of our knowledge, this is the first study to identify the association between SD and osteoporosis using meteorological data. We found that a lower occurrence of osteoporosis was associated with MHPA and long SD using Korean national cohort data with a matching method. Specifically, MHPA was associated with a lower occurrence of osteoporosis in various subgroups except >60-year-old male and underweight subgroups. On the other hand, long SD was associated with a lower occurrence of osteoporosis in females in the <60-year-old, LPA, obese, nonsmoker, or lower alcohol consumption subgroups.

Increasing evidence has demonstrated that PA is positively associated with BMD; hence, PA can prevent osteoporosis and fractures. One review study summarized 43 randomized controlled trial studies by conducting a meta-analysis and found that non-weight-bearing high-force exercise was the most effective exercise intervention for BMD of the femur neck (mean difference = 1.03, 95% CI = 0.24–1.82), and combination exercise programs were effective at improving spine BMD (mean difference = 3.22, 95% CI = 1.80–4.64) in postmenopausal women [12]. In one longitudinal study, men who engaged in weight-bearing exercise during their lifetime had a reduced risk of low bone strength in old age. In detail, the bone mineral content and inertia were 6.5–14.2% higher in the group that performed high-weight-bearing exercise in their lifetime than in the group that performed low-weight-bearing exercise in their lifetime [11]. One cohort study reported that 5-year mean PA with adjustment for BMI was associated with increased lumbar spine BMD in men (0.010 (95% CI = 0.004–0.017) g/cm^2^) and increased total hip BMD in women (0.006 (95% CI = 0.003–0.009) g/cm^2^) [13]. The results of our study that was conducted using a national sample cohort also support the above study findings. Moreover, the study findings were consistent in detailed and varied subgroups such as the obesity status, smoking, and alcohol consumption subgroups, but the same was not true for the young male and underweight subgroups.

To our knowledge, this is the first study to suggest that long SD is associated with a lower occurrence of osteoporosis using national cohort data. Although SD is a broad range of vitamin D indicator, the long SD group was associated with a lower occurrence of osteoporosis in our study. This finding is consistent with previous studies exploring the links of vitamin D obtained via dietary intake or supplementation, serum 25(OH)D, and sunlight exposure with BMD, osteoporosis, or fractures [20,21,23,33]. One experimental study using rats reported that PTH was significantly reduced in the sun-exposed vitamin D-deficient group (67.69 ± 13.18 pg/ml) compared to the levels in the vitamin D-supplemented rats (78.93 ± 8.31 pg/ml) and vitamin D-deficient rats (86.05 ± 9.67 pg/ml) [22]. Regarding sunlight exposure in humans, one study demonstrated that high BG grade as a biomarker of lifetime UV radiation had beneficial associations with reduced fracture prevalence in females (fewer major fractures in women with higher BG grade than lower BG grade, relative risk (RR) = 0.75, 95% CI = 0.60–0.97) [23]. Based on the above studies and our study findings, daily SD could be considered one of the factors affecting bone health.

Although PA × SD interaction was not significant (*p* = 282), the OR for osteoporosis was the lowest in the MHPA with long SD group compared to other combined PA and SD groups (reference = LPA with short SD, OR = 0.87, 95% CI = 0.83–0.91, Table S3). In other words, combining a higher intensity of PA with a longer SD could help to lower the risk of osteoporosis.

PA could improve bone strength through multiple mechanisms, such as gravitational loads and muscle contraction forces [34]. Specifically, gravitational loads occur when the weighted body makes contact with the surface. Without gravitational loads, for instance, astronauts in space could have increased risk of bone loss because of the absence of gravity [35]. In addition, muscle contraction forces could function in a particular region where a contraction was forced. For example, resistance training in athletes and cyclists was positively associated with BMD of the legs [36]. Furthermore, our study findings showed an association with various characteristics, such as female sex, older male sex, and other lifestyle factors, including SD, smoking status, and alcohol consumption. Based on these findings, MHPA could be recommended for the general population to improve bone health.

However, the association between PA and osteoporosis was not shown in the <60-year-old male subgroup or the underweight subgroup in our study. The common reason for these findings might be the lack of participants in each subgroup (the number of age < 60-year-old male group = 1314; the number of underweight group = 1655 out of a total of 58,053 participants). Another possible reason for the lack of an association in the <60-year-old male group is that the PA questionnaire did not classify PA into occupational PA and leisure PA. The retirement age in South Korea is usually 60 years old. A previous study using the same cohort data for PA and mortality analyses demonstrated that PA did not reduce mortality in males <60 years old. The PA in the study was not classified into occupational PA and leisure PA in the questionnaire, as described in our previous study [25]. In previous studies, in men with occupational PA, PA was not associated with BMD or was even associated with lower bone mineral content [37,38]. Another possible reason is that BMD is usually preserved or increased until <60 years old in men [37]. In summary, in young males, MHPA was not associated with the occurrence of osteoporosis in our study because occupational PA was not separated from leisure PA in the MHPA group, and osteoporosis among young males was rare.

Regarding the outcome of the underweight subgroup in our study, the previous study reported that active women were consistently at a lower risk of hip fracture than inactive women in each BMI category. However, the study finding shows that the risk for hip fracture was significantly higher in individuals with a low BMI than in individuals with a high BMI regardless of the intensity of PA [14]. Based on these findings and our study findings, both lower BMI and MHPA might affect BMD, making the association seem to disappear. On the other hand, previous studies emphasized that bone loss was more directly associated with loss of muscle mass than loss of fat mass. Moreover, weight loss resulting from exercising was not associated with bone loss [39,40]. Therefore, reduced BMI due to PA may not be the cause of bone loss.

The mechanism of vitamin D synthesis resulting from sun exposure in the body is as follows. When the skin is exposed to sunlight, UVB radiation interacts with 7-dehydrocholesterol and forms cholecalciferol (vitamin D3), which is the primary source of endogenous vitamin D [17,41]. Elevated serum 25(OH)D levels increase BMD by increasing the absorption of calcium and phosphorus from the intestine by reducing PTH, preventing osteoporosis [16,17]. These mechanisms could explain our study findings regarding the association between SD and the low occurrence of osteoporosis. Moreover, females in the <60-year-old, LPA, obese, nonsmoker, and lower alcohol consumption subgroups showed the same results.

Regarding the outcome in the < 60-year-old female subgroup in our study, a previous cross-sectional study reported that compared to the LPA with low serum 25(OH)D group, the low PA with high serum 25(OH)D group had a lower prevalence of osteoporosis among females [15]. Females who were ≥ 60 years old did not exhibit this association in our study possibly because bone loss may have already started due to the effect of estrogen deficiency regarding menopause [42,43]. Another possible reason is that the SD did not accurately represent the real effect of vitamin D so that other subgroups of age and sex might not have exhibited the associations.

Regarding LPA and the association between SD and osteoporosis, a cross-sectional study also reported that the odds ratio of the low PA with high 25(OH)D group for osteoporosis was significantly lower than that of the low PA with low 25(OH)D group [15]. In other words, the LPA group could have improved their bone health by increasing their vitamin D status through increased sun exposure even though PA is not available.

In addition, long SD was inversely associated with the risk of osteoporosis in the obese, nonsmoker, and lower alcohol consumption (<1 time a week) subgroups in our study. Obesity has a positive effect on bone health [14], whereas smoking and alcohol consumption negatively affect bone health [44,45]. In this regard, long SD may be more effective at decreasing the occurrence of osteoporosis when individuals have other positive characteristics associated with increased BMD. Further studies are needed to elucidate the effect of SD on bone health when participants have positive characteristics associated with improved bone health.

The main strength of our study was the use of a large cohort with a 7-year follow-up period. In addition, we matched the osteoporosis and control groups at a 1:2 ratios for age, sex, income, and region of residence to identify the independent association between osteoporosis and the intensity of PA and between osteoporosis and SD. Furthermore, subgroup analyses according to age, sex, PA, SD, obesity, smoking status, and alcohol consumption status were performed to evaluate whether the findings were consistent in individuals with different characteristics.

Several limitations remained in our study. The major limitation is that the study used secondary data. Accordingly, we could not consider the intake of vitamin D or calcium. Moreover, the METs could not be correctly calculated because the questionnaire did not ask for a specific PA duration, which is similar to the data used in a previous study [25]. Instead, PA groups were classified into only LPA and MHPA. In addition, PA was not specifically classified according to whether it was performed during leisure or occupational time. Another limitation is that SD did not reflect the data of individual sunlight exposure. Finally, the causal relationship between PA/SD and osteoporosis is not definite because of the limitation of the observational study design.

## 5. Conclusions

We suggest that both MHPA and long SD are inversely associated with osteoporosis. Specifically, MHPA might decrease the occurrence of osteoporosis in individuals with various characteristics or lifestyles, such as age/sex background, SD, obesity status, smoking status, and alcohol consumption status, but this might not be true for young males or underweight individuals. Long SD might decrease the occurrence of osteoporosis in young females, obese individuals, nonsmokers, and individuals with lower alcohol consumption. Therefore, we concluded that outdoor PA, which could not only increase the intensity of PA but also expose people to the sun for a long time, might be beneficial for preventing osteoporosis.

## Figures and Tables

**Table 1:** General Characteristics of Participants.

Characteristics	Total Participants		
	Osteoporosis (*n*, %)	Control (*n*, %)	*p*-Value
Age (years old)			1.000
45–49	205 (1.1)	410 (1.1)	
50–54	2297 (11.9)	4594 (11.9)	
55–59	3028 (15.7)	6056 (15.7)	
60–64	3243 (16.8)	6486 (16.8)	
65–69	3575 (18.5)	7150 (18.5)	
70–74	3854 (19.9)	7708 (19.9)	
75–79	2126 (11.0)	4252 (11.0)	
80–84	819 (4.2)	1638 (4.2)	
85+	204 (1.1)	408 (1.1)	
Sex			1.000
Male	3711 (19.2)	7422 (19.2)	
Female	15,640 (80.8)	31,280 (80.8)	
Income			1.000
1 (lowest)	3425 (17.7)	6850 (17.7)	
2	2694 (13.9)	5388 (13.9)	
3	3162 (16.3)	6324 (16.3)	
4	4015 (20.8)	8030 (20.8)	
5 (highest)	6055 (31.3)	12,110 (31.3)	
Region of residence			1.000
Urban	7201 (37.2)	14,402 (37.2)	
Rural	12,150 (62.8)	24,300 (62.8)	
Obesity †			<0.001 *
Underweight	738 (3.8)	917 (2.4)	
Normal	7619 (39.4)	13,074 (33.8)	
Overweight	4999 (25.8)	10,189 (26.3)	
Obese I	5463 (28.2)	12,815 (33.1)	
Obese II	532 (2.8)	1707 (4.4)	
Smoking status			0.029 *
Nonsmoker	17,003 (87.9)	33,736 (87.2)	
Past smoker	1370 (7.1)	2824 (7.3)	
Current smoker	978 (5.1)	2142 (5.5)	
Alcohol consumption			<0.001 *
<1 time a week	14,965 (77.3)	29,254 (75.6)	
≥1 time a week	4386 (22.7)	9448 (24.4)	
CCI score			<0.001 *
0	13,083 (67.6)	27,493 (71.0)	
1	3043 (15.7)	5453 (14.1)	
≥2	3225 (16.7)	5756 (14.9)	
PA			<0.001 *
LPA	11,209 (57.9)	21,462 (55.5)	
MHPA	8142 (42.1)	17,240 (44.6)	
Sunshine duration			0.005 *
Short sunshine duration (≤6 h)	9625 (49.7)	18,772 (48.5)	
Long sunshine duration (>6 h)	9726 (50.3)	19,930 (51.5)	

Abbreviations: CCI, Charlson comorbidity index; LPA, low-intensity physical activity; MHPA, moderate- to high-intensity physical activity; PA, physical activity.* Chi-square test. Significance at *p* < 0.05. † Obesity (BMI, body mass index, kg/m^2^) was categorized as <18.5 (underweight), ≥18.5 to <23 (normal), ≥23 to <25 (overweight), ≥25 to <30 (obese I), and ≥30 (obese II).

**Table 2:** Crude and Adjusted Odds Ratios (95% Confidence Intervals) of Moderate- to High-Intensity Physical Activity for Osteoporosis with Subgroup Analyses According to Age and Sex.

Characteristics	No. of Osteoporosis/No. of Participants (%)	Odds Ratios for Osteoporosis			
		Crude †	*p*-Value	Adjusted †‡	*p*-Value
Total participants (*n* = 58,053)					
MHPA	8142/25,382 (32.1)	0.90 (0.87–0.94)	<0.001 *	0.90 (0.87–0.94)	<0.001 *
LPA	11,209/32,671 (34.3)	1.00		1.00	
Age < 60 years old, males (*n* = 1314)					
MHPA	194/613 (31.7)	0.87 (0.69–1.09)	0.223	0.88 (0.69–1.12)	0.307
LPA	244/701 (34.8)	1.00		1.00	
Age < 60 years old, females (*n* = 15,276)					
MHPA	2158/6,637 (32.5)	0.94 (0.88–1.00)	0.059	0.93 (0.87–1.00)	0.048 *
LPA	2934/8,639 (34.0)	1.00		1.00	
Age ≥ 60 years old, males (*n* = 9819)					
MHPA	1524/4,945 (30.8)	0.79 (0.73–0.86)	<0.001 *	0.81 (0.74–0.88)	<0.001 *
LPA	1,749/4,874 (35.9)	1.00		1.00	
Age ≥ 60 years old, females (*n* = 31,644)					
MHPA	4266/13,187 (32.4)	0.93 (0.88–0.97)	0.002 *	0.92 (0.88–0.96)	0.001 *
LPA	6282/18,457 (34.0)	1.00		1.00	

Abbreviations: CCI, Charlson comorbidity index; LPA, low-intensity physical activity; MHPA, moderate- to high-intensity physical activity.* Conditional logistic regression, Significance at *p* < 0.05. † Models stratified by age, sex, income, and region of residence. ‡ Adjusted for sunshine duration, obesity, smoking, alcohol consumption, and CCI scores.

**Table 3:** Crude and Adjusted Odds Ratios (95% Confidence Intervals) of Long Sunshine Duration for Osteoporosis with Subgroup Analyses According to Age and Sex.

Characteristics	No. of Osteoporosis/No. of Participants (%)	Odds Ratios for Osteoporosis			
		Crude†	*p*-Value	Adjusted †‡	*p*-Value
Total participants (*n* = 58,053)					
Long sunshine duration	9726/29,656 (32.8)	0.95 (0.92–0.98)	0.004 *	0.96 (0.93–1.00)	0.027 *
Short sunshine duration	9625/28,397 (33.9)	1.00		1.00	
Age < 60 years old, males (*n* = 1314)					
Long sunshine duration	233/717 (32.5)	0.91 (0.71–1.16)	0.450	0.94 (0.73–1.21)	0.605
Short sunshine duration	205/597 (34.3)	1.00		1.00	
Age < 60 years old, females (*n* = 15,276)					
Long sunshine duration	2489/7,631 (32.6)	0.93 (0.87–1.00)	0.050	0.93 (0.87–1.00)	0.045 *
Short sunshine duration	2603/7645 (34.1)	1.00		1.00	
Age ≥ 60 years old, males (*n* = 9819)					
Long sunshine duration	1675/5,118 (32.7)	0.94 (0.86–1.03)	0.165	0.98 (0.90–1.07)	0.623
Short sunshine duration	1598/4,701 (34.0)	1.00		1.00	
Age ≥ 60 years old, females (*n* = 31,644)					
Long sunshine duration	5329/16,190 (32.9)	0.96 (0.91–1.01)	0.095	0.96 (0.92–1.01)	0.136
Short sunshine duration	5219/15,454 (33.8)	1.00		1.00	

Abbreviations: CCI, Charlson comorbidity index; PA, physical activity.* Conditional logistic regression, Significance at *p* < 0.05. † Models stratified by age, sex, income, and region of residence. ‡ Adjusted for PA, obesity, smoking, alcohol consumption, and CCI scores.

## Data Availability

Restrictions apply to the availability of these data. Data were obtained from the Korean National Health Insurance Sharing Service (NHISS) and are available at https://nhiss.nhis.or.kr (accessed on 21 April 2021) with the permission of NHIS.

## Supplementary Materials

The following are available online at https://www.mdpi.com/article/10.3390/ijerph18094437/s1, Table S1: Subgroup analyses of crude and adjusted odds ratios (95% confidence intervals) of moderate- to high-intensity physical activity for osteoporosis according to sunshine duration, obesity, smoking, and alcohol consumption. Table S2: Subgroup analyses of crude and adjusted odds ratios (95% confidence intervals) of long sunshine duration for osteoporosis according to physical activity, obesity, smoking, and alcohol consumption. Table S3: Crude and adjusted odds ratios (95% confidence intervals) for osteoporosis in PA × sunshine duration interaction, and combined PA and sunshine duration group.
