# Supplementary material for: Associations between Physical Activity, Sunshine Duration and Osteoporosis According to Obesity and Other Lifestyle Factors: A Nested Case–Control Study
Source: Int J Environ Res Public Health. 2021 Apr 22;18(9):4437. doi: 10.3390/ijerph18094437 (PMC8122401; doi:10.3390/ijerph18094437)
Supplement: Supplementary file 1 [file ijerph-18-04437-s001.zip › ijerph-1175890-supplementary.docx]

**Table S1 Subgroup analyses of crude and adjusted odds ratios (95% confidence intervals) of moderate- to high-intensity physical activity for osteoporosis according to sunshine duration, obesity, smoking, and alcohol consumption.**

| **Characteristics** | | **No. of osteoporosis /No. of participants (%)** | | **Odds ratios for osteoporosis** | | | |
| --- | --- | --- | --- | --- | --- | --- | --- |
|  |  |  | | **Model 1†** | ***p*-value** | **Model 2‡** | ***p*-value** |
| **Short sunshine duration (n = 28,397)** | | |  | | | | |
| MHPA | | 3,837/11,873 (32.3) | | 0.89 (0.84-0.93) | <0.001* | 0.89 (0.85-0.94) | <0.001* |
| LPA | | 5,788/16,524 (35.0) | | 1.00 |  | 1.00 |  |
| **Long sunshine duration (n = 29,656)** | | |  | | | | |
| MHPA | | 4,305/13,509 (31.9) | | 0.92 (0.88-0.97) | 0.001* | 0.92 (0.87-0.97) | 0.001* |
| LPA | | 5,421/16,147 (33.6) | | 1.00 |  | 1.00 |  |
| **Underweight (n = 1,655)** | | |  | | | | |
| MHPA | | 308/661 (46.6) | | 1.08 (0.89-1.33) | 0.434 | 1.09 (0.89-1.33) | 0.422 |
| LPA | | 430/994 (43.3) | | 1.00 |  | 1.00 |  |
| **Normal weight (n = 20,693)** | | |  | | | | |
| MHPA | | 3,229/9,077 (35.6) | | 0.90 (0.85-0.96) | 0.001* | 0.90 (0.85-0.96) | 0.001* |
| LPA | | 4,390/11,616 (37.8) | | 1.00 |  | 1.00 |  |
| **Overweight (n = 15,188)** | | |  | | | | |
| MHPA | | 2,157/6,913 (31.2) | | 0.87 (0.81-0.93) | <0.001* | 0.87 (0.81-0.93) | <0.001* |
| LPA | | 2,842/8,275 (34.3) | | 1.00 |  | 1.00 |  |
| **Obese (n = 20,517)** | | |  | | | | |
| MHPA | | 2,448/8,731 (28.0) | | 0.92 (0.86-0.97) | 0.005* | 0.92 (0.86-0.98) | 0.008* |
| LPA | | 3,547/11,786 (30.1) | | 1.00 |  | 1.00 |  |
| **Nonsmoker (n = 50,739)** | | |  | | | | |
| MHPA | | 7,077/21,788 (32.5) | | 0.92 (0.89-0.96) | <0.001* | 0.92 (0.89-0.96) | <0.001* |
| LPA | | 9,926/28,951 (34.3) | | 1.00 |  | 1.00 |  |
| **Past smoker and current smoker (n = 7,314)** | | |  | | | | |
| MHPA | | 1,065/3,594 (29.6) | | 0.79 (0.71-0.87) | <0.001* | 0.80 (0.72-0.89) | <0.001* |
| LPA | | 1,283/3,720 (34.5) | | 1.00 |  | 1.00 |  |
| **< 1 time a week of alcohol consumption (n = 44,219)** | | |  | | | | |
| MHPA | | 6,119/18,689 (32.7) | | 0.91 (0.88-0.95) | <0.001* | 0.91 (0.88-0.95) | <0.001* |
| LPA | | 8,846/25,530 (34.7) | | 1.00 |  | 1.00 |  |
| **≥ 1 time a week of alcohol consumption (n = 13,834)** | | |  | | | | |
| MHPA | | 2,023/6,693 (30.2) | | 0.88 (0.82-0.95) | 0.001* | 0.88 (0.82-0.95) | 0.001* |
| LPA | | 2,363/7,141 (33.1) | | 1.00 |  | 1.00 |  |

Abbreviations: CCI, Charlson comorbidity index; LPA, low-intensity physical activity; MHPA, moderate- to high-intensity physical activity. * Un-conditional logistic regression, Significance at *p* < 0.05. † A model 1 was adjusted for age, sex, income, and region of residence. ‡ A model 2 was adjusted for model 1 plus sunshine duration, obesity, smoking, alcohol consumption, and CCI scores.

**Table S2 Subgroup analyses of crude and adjusted odds ratios (95% confidence intervals) of long sunshine duration for osteoporosis according to physical activity, obesity, smoking, and alcohol consumption.**

| **Characteristics** | | **No. of osteoporosis /No. of participants (%)** | | **Odds ratios for osteoporosis** | | | |
| --- | --- | --- | --- | --- | --- | --- | --- |
|  |  |  | | **Model 1†** | ***p*-value** | **Model 2‡** | ***p*-value** |
| **LPA (n = 32,671)** | | |  | | | | |
| Long sunshine duration | | 5,421/16,147 (33.6) | | 0.94 (0.90-0.99) | 0.011* | 0.95 (0.91-1.00) | 0.047* |
| Short sunshine duration | | 5,788/16,524 (35.0) | | 1.00 |  | 1.00 |  |
| **MHPA (n = 25,382)** | | |  | | | | |
| Long sunshine duration | | 4,305/13,509 (31.9) | | 0.96 (0.91-1.02) | 0.189 | 0.98 (0.92-1.03) | 0.365 |
| Short sunshine duration | | 3,837/11,873 (32.3) | | 1.00 |  | 1.00 |  |
| **Underweight (n = 1,655)** | | |  | | | | |
| Long sunshine duration | | 373/833 (44.8) | | 1.00 (0.81-1.22) | 0.976 | 1.00 (0.81-1.23) | 0.998 |
| Short sunshine duration | | 365/822 (44.4) | | 1.00 |  | 1.00 |  |
| **Normal weight (n = 20,693)** | | |  | | | | |
| Long sunshine duration | | 3,878/10,609 (36.6) | | 0.97 (0.92-1.03) | 0.363 | 0.99 (0.94-1.05) | 0.800 |
| Short sunshine duration | | 3,741/10,084 (37.1) | | 1.00 |  | 1.00 |  |
| **Overweight (n = 15,188)** | | |  | | | | |
| Long sunshine duration | | 2,573/7,828 (32.9) | | 1.00 (0.93-1.07) | 0.893 | 1.00 (0.94-1.08) | 0.906 |
| Short sunshine duration | | 2,426/7,360 (33.0) | | 1.00 |  | 1.00 |  |
| **Obese (n = 20,517)** | | |  | | | | |
| Long sunshine duration | | 2,092/10,386 (27.9) | | 0.88 (0.83-0.93) | <0.001* | 0.89 (0.84-0.95) | <0.001* |
| Short sunshine duration | | 3,093/10,131 (30.5) | | 1.00 |  | 1.00 |  |
| **Nonsmoker (n = 50,739)** | | |  | | | | |
| Long sunshine duration | | 8,449/25,669 (32.9) | | 0.95 (0.91-0.98) | 0.003* | 0.96 (0.92-0.99) | 0.020* |
| Short sunshine duration | | 8,554/25,070 (34.1) | | 1.00 |  | 1.00 |  |
| **Past smoker and current smoker (n = 7,314)** | | |  | | | | |
| Long sunshine duration | | 1,277/3,987 (32.0) | | 0.99 (0.89-1.09) | 0.792 | 1.02 (0.92-1.13) | 0.738 |
| Short sunshine duration | | 1,071/3,327 (32.2) | | 1.00 |  | 1.00 |  |
| **< 1 time a week of alcohol consumption (n = 44,219)** | | |  | | | | |
| Long sunshine duration | | 6,992/21,094 (33.2) | | 0.94 (0.90-0.97) | 0.001* | 0.94 (0.90-0.98) | 0.003* |
| Short sunshine duration | | 7,973/23,125 (34.5) | | 1.00 |  | 1.00 |  |
| **≥ 1 time a week of alcohol consumption (n = 13,834)** | | |  | | | | |
| Long sunshine duration | | 2,734/8,562 (31.9) | | 1.04 (0.96-1.12) | 0.365 | 1.04 (0.96-1.12) | 0.333 |
| Short sunshine duration | | 1,652/5,272 (31.3) | | 1.00 |  | 1.00 |  |

Abbreviations: CCI, Charlson comorbidity index; LPA, low-intensity physical activity; MHPA, moderate- to high-intensity physical activity; PA, physical activity. * Un-conditional logistic regression, Significance at *p* < 0.05. † A model 1 was adjusted for age, sex, income, and region of residence. ‡ A model 2 was adjusted for model 1 plus PA, obesity, smoking, alcohol consumption, and CCI scores.

**Table S3 Crude and adjusted odds ratios (95% confidence intervals) for osteoporosis in PA** **× sunshine duration interaction, and combined PA and sunshine duration group.**

| **Characteristics** | | **No. of osteoporosis/** | **Odds ratios for osteoporosis** | |
| --- | --- | --- | --- | --- |
|  |  | **No. of participants (%)** | **Adjusted†** | ***p*-value** |
| **PA** **× sunshine duration‡** | | NA | 1.04 (0.97-1.01) | 0.282 |
| **Combined PA and sunshine duration group§** | |  |  |  |
| MHPA + long sunshine duration | | 4,305/13,509 (31.9) | 0.87 (0.83-0.91) | <0.001* |
| MHPA + short sunshine duration | | 3,837/11,873 (32.3) | 0.89 (0.84-0.93) | <0.001* |
| LPA + long sunshine duration | | 5,421/16,147 (33.6) | 0.94 (0.90-0.99) | 0.017* |
| LPA + short sunshine duration (reference) | | 5,788/16,524 (35.0) | 1.00 |  |

Abbreviations: CCI, Charlson comorbidity index; LPA, low-intensity physical activity; MHPA, moderate- to high-intensity physical activity, NA, not applicable. * Conditional logistic regression, Significance at *p* < 0.05. † Stratified by age, sex, income, and region of residence. ‡ Adjusted for PA, sunshine duration, PA × sunshine duration, obesity, smoking, alcohol consumption, and CCI scores. § Adjusted for obesity, smoking, alcohol consumption, and CCI scores.
